# wDBTF: an integrated database resource for studying wheat transcription factor families

**DOI:** 10.1186/1471-2164-11-185

**Published:** 2010-03-18

**Authors:** Isabelle Romeuf, Dominique Tessier, Mireille Dardevet, Gérard Branlard, Gilles Charmet, Catherine Ravel

**Affiliations:** 1 Institut National de la Recherche Agronomique (INRA), UMR1095, Génétique, Diversité et Ecophysiologie des Céréales, 234 avenue du Brézet, Clermont-Ferrand, F-63100 France; Université Blaise-Pascal, UMR1095, Campus des Cézeaux, F-63170 Aubière, France; 2 Institut National de la Recherche Agronomique (INRA), UR Biopolymères, Interactions, Assemblages (BIA), rue de la Géraudière, Nantes, F-44316, France

## Abstract

**Background:**

Transcription factors (TFs) regulate gene expression by interacting with promoters of their target genes and are classified into families based on their DNA-binding domains. Genes coding for TFs have been identified in the sequences of model plant genomes. The rice (*Oryza sativa *spp. *japonica*) genome contains 2,384 TF gene models, which represent the mRNA transcript of a locus, classed into 63 families.

**Results:**

We have created an extensive list of wheat (*Triticum aestivum *L) TF sequences based on sequence homology with rice TFs identified and classified in the Database of Rice Transcription Factors (DRTF). We have identified 7,112 wheat sequences (contigs and singletons) from a dataset of 1,033,960 expressed sequence tag and mRNA (ET) sequences available. This number is about three times the number of TFs in rice so proportionally is very similar if allowance is made for the hexaploidy of wheat. Of these sequences 3,820 encode gene products with a DNA-binding domain and thus were confirmed as potential regulators. These 3,820 sequences were classified into 40 families and 84 subfamilies and some members defined orphan families. The results were compiled in the Database of Wheat Transcription Factor (wDBTF), an inventory available on the web http://wwwappli.nantes.inra.fr:8180/wDBFT/. For each accession, a link to its library source and its Affymetrix identification number is provided. The positions of Pfam (protein family database) motifs were given when known.

**Conclusions:**

wDBTF collates 3,820 wheat TF sequences validated by the presence of a DNA-binding domain out of 7,112 potential TF sequences identified from publicly available gene expression data. We also incorporated *in silico *expression data on these TFs into the database. Thus this database provides a major resource for systematic studies of TF families and their expression in wheat as illustrated here in a study of DOF family members expressed during seed development.

## Background

Gene expression at the level of mRNA transcription from DNA is regulated through different mechanisms, the most widely studied being regulation by transcription factors (TFs). These DNA-binding proteins control gene expression by interacting with *cis*-regulatory elements (CREs) in the promoters of their target genes. DNA-binding domains are in general highly conserved among species so their characteristics are used to classify TFs into families. Transcriptional control of gene expression influences many biological processes such as responses to the environment or stress and regulation of metabolic pathways.

The sequencing of model plant genomes has allowed many genes coding for TFs to be identified. A total of 1,533 TFs were identified in the *Arabidopsis thaliana *genome [1] and classified into 34 families. Comparing these with TF genes in other eukaryotic genomes, i.e., of Drosophila, *Caenorhabditis elegans*, and *Saccharomyces cerevisiae*, the size of TF families differs considerably across the eukaryote kingdoms and about 45% of Arabidopsis TFs are from families specific to plants [2]. In *Oryza sativa *spp. *japonica*, Xiong et al. [3] identified 1,611 TF genes belonging to 37 gene families. Most TF families are of similar size in rice and in Arabidopsis [3].

Information on the TFs of model species has been organized into databases. Arabidopsis TF sequences were compiled and classified into families by several groups: the Riechmann group, the Sheen group http://genetics.mgh.harvard.edu/sheenweb/AraTRs.html, OHIO-ATTFDB [4], RARTF http://rarge.gsc.riken.jp/rartf/, TRANSFAC [5] and TrSDB [6]. Another Arabidopsis TF database (DATF, http://datf.cbi.pku.edu.cn/) was created that integrates information from multiple sources (expressed sequence tags (ESTs), transcription factor binding sites) and contains 1,922 known or predicted TF sequences, classified in 64 families [7]. The same approach was taken for both *O. sativa *spp. *japonica *and spp. *indica *to create DRTF http://drtf.cbi.pku.edu.cn/, which contains 2,384 rice TF sequences classified in 63 families [8]. The discrepancies in the total number of TFs included in databases and the classification into families for the same species depends on the methods used for predicting TFs. For example, the differences between the dataset in DRTF and the results of Xiong et al. [3] are mostly because of new annotation in the latest version (Release 4) of the TIGR database that uses a broader definition of TFs. The *sensu stricto *definition of TFs is based on the DNA binding domain, but in the broader sense proteins having co-activator activity can also be considered as TFs. This broader definition explains why more TF families (63 versus 37) were found more recently.

Wheat is the most important crop in world food production. *Triticum aestivum *and other cereal genomes are substantially larger than those of *A. thaliana *and *O. sativa*. The genome of hexaploid wheat is about 16,000 Mb which is 38 times larger than that of the monocotyledon model *O. sativa*. It is an allohexaploid composed of three homoeologous subgenomes, AA, BB, and DD, thus generally each gene is represented by three homoeologous copies. The large hexaploid nature of the genome is a drawback in determining the complete sequence which is not yet available. Nevertheless, many wheat cDNA libraries have been constructed generating a rich source of ESTs; more than one million wheat ESTs are currently available in dbEST [9]. These ESTs have been assembled into contigs such as Tentative consensus (TC) contigs ([10]; http://compbio.dfci.harvard.edu/cgi-bin/tgi/gimain.pl?gudb=Wheat) along with data collated from the NCBI GenBank nucleotide database (full length and partial cDNAs). ESTs directly represent the transcribed portions of the genome so while waiting for the full genome to be sequenced, the analysis of expressed sequences is a good starting point for gene discovery.

Recently some *T. aestivum *TF families have been described such as MIKC-type MADS-box [11], NF-Y and Dr1 [12], Myb [13] and the plant-specific DOF (DNA-binding with One Finger) gene families [14,15]. The DNA-binding domain of DOF proteins contains 52 amino acids structured as a Cys2/Cys2 zinc finger [16]. This domain recognizes and binds a core motif 5'-AAAG-3' on gene promoters [17]. In the Arabidopsis and rice genomes 36 and 30 Dof genes have been identified, respectively, and phylogenetic relationships between the genes have been established such that four major orthologous clusters have been defined [1,15]. Recently, 26 different Dof genes were identified in barley by sequence analysis of clones isolated by screening genomic libraries and ESTs [18]. By analysing soybean ESTs, 39 putative unigenes that encode DOF proteins were identified [19], 27 of which were confirmed as having the highly conserved domain [20].

DOF TFs participate in the regulation of many processes exclusive to plants. For example in barley, DOF TFs are involved at several developmental stages in seeds, in the accumulation of storage proteins and in germination [21-23]. In wheat, two Dof genes have been reported: wheat prolamin-box binding factor (*WPBF*), shown to be an activator of *T. aestivum *L. storage protein genes [24], and *TaDof1 *[25]. The three homoeologous genes encoding WPBF were sequenced and shown to be specifically expressed in seeds between 3 and 39 days after anthesis [26].

The aim of this study was to identify all unique *T. aestivum *TFs (i.e. each homoeolog is considered independently) by using generally available wheat transcript sequences and the model plant rice genome. We constructed a database compiling the wheat TF sequences identified, the corresponding *in silico *expression data and the predicted DNA-binding domains. The study of DOF family members illustrates the utility of wDBTF and the overall analytical approach.

## Construction and content

### Source data and database construction

**Figure 1:**
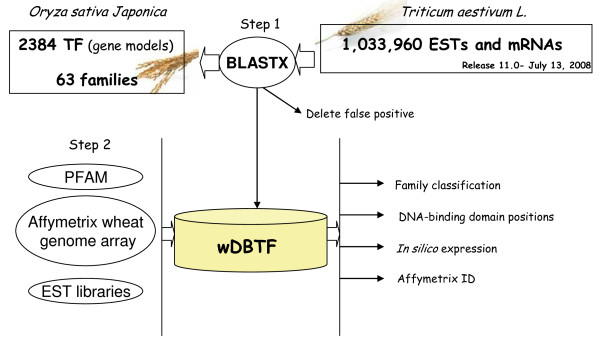
**Schematic representation of the wDBTF wheat transcription factor database**. The core database contains rice gene models from DRTF. The wheat data is made up of publicly available expressed sequences either singletons or assembled contigs. wDBTF includes information on 7,112 identified wheat TFs, on the DNA-binding domains (where known) and on *in silico *expression.

The 2,384 putative TF protein sequences of *O. sativa japonica *were downloaded from the DRTF database http://drtf.cbi.pku.edu.cn/. *T. aestivum *sequences were downloaded from the Gene Indices database (wheat latest release 11.0, http://compbio.dfci.harvard.edu/tgi/plant.html) which represents 1,036,933 sequences either as TC contigs or singletons. The wheat sequences were translated to peptide sequences and compared using the BlastX program [27] with *O. sativa japonica *TF proteins. We used an e-value cut-off of 1e-05 with other parameters at the default setting, keeping only the best Blast hit. As TFs are composed of several conserved domains, some BlastX results gave a high Blast score against a rice TF protein because of particular conserved domains but were not really related to the query TF. To avoid such false associations, all Blast results were checked by eye and the database was edited to remove 2,296 sequences. The wheat TF sequences identified were compiled in the wDBTF database (Figure 1).

The amino acid sequences from *T. aestivum *were deduced from BlastX alignment against *O. sativa *proteins using the method described by Sado et al. [28]. Only the protein sequence regions that matched a TF sequence were taken into account. The presence of DNA-binding domain for each wheat TF sequence identified was searched against the Pfam database. The e-value cut-off 1e-01 was used in the initial Hidden Markov Model (HMMer) searches. For families which have no DNA-binding domain profiles available in the Pfam database, each sequence was analyzed individually to decide the cut-off value and sequences with more than 60% identity with their respective sequences from rice were considered to be *bona fide *members.

The Affymetrix wheat genome array contains 61,127 probe sets which represent 55,052 transcripts. Blast searches were performed against the probe set data to assign an Affymetrix ID to individual sequences.

**Figure 2:**
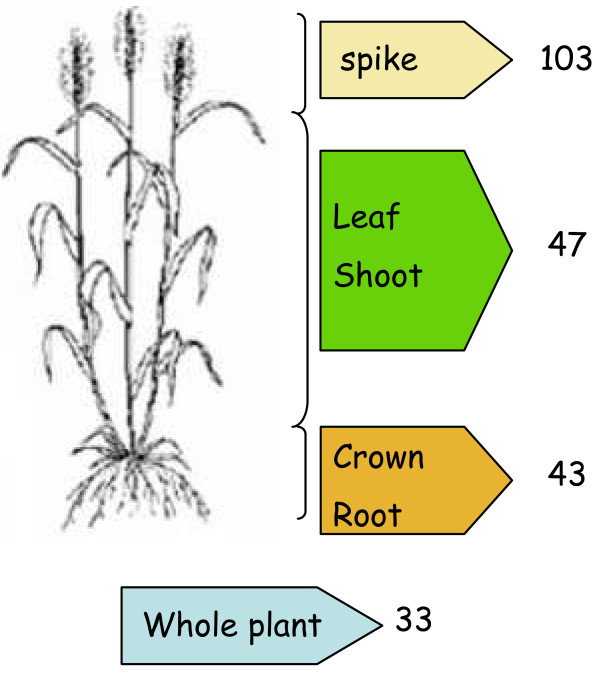
**Categories of the 226 cDNA libraries used to identify wheat TFs**. Spike origin refers to libraries from flower, spikelet, seed or entire spike tissue.

Details of the all the original EST libraries were collated from the NCBI Unigene site http://www.ncbi.nlm.nih.gov/unigene. The list of 226 libraries used for this database is provided in Additional file 1. The origins of expressed sequences were classified in six categories: root, crown, shoot, leaf, whole plant if the origin is unknown, and spike for whole organs and tissues within the spike at different developmental stages from flowering to maturation (Figure 2).

All data were imported and formatted using custom PERL scripts and stored in a PostgreSQL database, named wDBTF, publicly available at http://wwwappli.nantes.inra.fr:8180/wDBFT/. The web site was developed with Java applet and servlet technologies. The wDBTF web pages are conceptual views of the PostgreSQL relational database.

### Identification of TFs in *T. aestivum*

Comparative analysis of wheat and rice TFs based on sequence identity was performed to identify all unique TFs in public transcript sequences of wheat.

Each of the 216,452 unique wheat sequences (contigs and singletons) from the TIGR assembly of ESTs and transcripts was systematically compared with rice TF proteins using Blastx. The sequences matching rice TFs (e-value < 1e-05) were defined as TF-encoding sequences. Any sequences which did not correspond to potential TFs were discarded. In this way we found 7,112 accessions from TIGR correspond to *T. aestivum *TFs. These sequences were represented by 3,790 TCs and 3,322 singletons (Table 1). Evidence of a conserved DNA-binding domain was detected for 51.8% of the wheat sequences identified, i.e. 3,681 sequences (Table 1). For 8 (GRF, LUG, NZZ, Trihelix, ULT, Whirly, HRT-like and VOZ) out of 84 subfamilies, no DNA-binding domain motifs are available in the Pfam database, so we established whether a wheat sequence belongs to a specific TF subfamily according to its degree of similarity to the respective subfamily member from rice. After this step 3,820 sequences were found to correspond to *bona fide *wheat TFs as they have the DNA-binding domain required to validate their status as transcriptional regulators.

**Table 1:** Number of genes belonging to TF families identified in rice and wheat.

		***O.sativa japonica***	***T.aestivum***				
			
**Nb**	**SubFamily name**	**gene models**	**TCs^a^**	**ESTs^b^**	**singletons**	**Total^c^**	**TF^d^**
		
1	AP2	28	26	192	21	47	30
2	EREBP	148	199	1457	214	413	265
3	RAV	6	15	132	10	25	18
4	ARID	7	15	104	7	22	11
5	AS2	39	16	97	24	40	31
6	AUX/IAA	46	104	813	114	218	200
7	ARF	43	72	504	66	138	37
8	LAV	11	12	92	12	24	7
9	RAV	8	5	27	4	9	4
10	REM	36	57	265	54	111	87
11	BBR/BPC	7	3	15	6	9	9
12	BES1	6	11	78	5	16	8
13	bHLH	184	243	1153	225	468	207
14	bZIP	109	188	1230	139	327	167
15	CAMTA	8	23	168	18	41	25
16	TAZ	10	43	230	21	64	20
17	CCAAT-Dr1	1	6	42	3	9	8
18	CCAAT-HAP2	20	25	139	13	38	22
19	CCAAT-HAP3	17	21	110	14	35	24
20	CCAAT-HAP5	18	17	152	16	33	26
21	CPP	16	12	117	13	25	10
22	E2F/DP	9	16	46	23	39	20
23	EIL	12	15	141	20	35	22
24	FHA	19	45	313	29	74	32
25	GARP-ARR-B	11	15	106	16	31	5
26	GARP-G2-like	56	88	478	66	154	73
27	GeBP	15	11	73	13	24	4
28	GIF	3	5	55	12	17	12
29	DELLA	4	5	49	10	15	14
30	HAM	10	5	28	14	19	14
31	LISCL	15	15	84	18	33	24
32	LS	3	0	0	2	2	1
33	PAT1	8	31	263	20	51	38
34	SCL3	9	0	0	2	2	2
35	SCR	4	2	16	3	5	4
36	SHR	5	1	2	3	4	3
37	GRF	18	20	109	8	28	28
38	BHL	14	16	100	21	37	4
39	HD-ZIP	62	116	625	89	205	72
40	KNOX	8	19	104	11	30	18
41	PHD	2	12	66	2	14	5
42	WOX	17	9	30	6	15	4
43	ZF-HD	15	8	43	11	19	14
44	HMG	19	89	2350	67	156	133
45	HSF	36	48	180	46	94	56
46	JUMONJI	17	31	174	27	58	20
47	LFY	1	1	3	1	2	2
48	LUG	11	20	125	18	38	26
49	M-type	23	1	4	2	3	2
50	MIKC-type	60	127	1328	57	184	128
51	MBF1	3	31	688	46	77	42
52	R1R2R3	5	7	14	6	13	5
53	R2R3	133	164	702	165	329	203
54	MYB-related	84	159	1082	114	273	116
55	NAC	149	188	1226	193	381	269
56	Nin-like	14	16	71	14	30	14
57	NZZ	1	0	0	3	3	3
58	ASH1	6	12	61	13	25	9
59	EZ	2	7	46	8	15	3
60	RBCMT	8	16	187	26	42	23
61	SUVAR	15	44	266	38	82	51
62	TRX	3	19	106	5	24	10
63	S1Fa-like	4	11	479	12	23	23
64	TCP	24	19	58	11	30	14
65	Trihelix	23	36	175	21	57	57
66	TUB	21	41	308	36	77	63
67	ULT	2	1	9	0	1	1
68	Whirly	1	5	58	6	11	11
69	Alfin	13	25	196	24	49	25
70	C2C2-CO-like	54	119	820	120	239	81
71	C2C2-DOF	36	25	148	34	59	28
72	C2C2-GATA	23	47	221	31	78	25
73	C2C2-YABBY	12	20	303	11	31	30
74	C2H2	113	139	975	123	262	122
75	C3H	90	169	1378	129	298	169
76	HRT-like	1	2	6	1	3	3
77	LIM	13	37	591	36	73	39
78	PHD	79	243	1586	170	413	126
79	PLATZ	20	25	185	20	45	33
80	SBP	28	21	160	23	44	15
81	SRS	6	0	0	1	1	1
82	VOZ	2	7	36	3	10	10
83	ZIM	29	95	1076	86	181	13
84	WRKY	113	156	687	177	333	187
			
**Total**		2384	3790	27916	3322	7112	3820

^a ^number of tentative consensus contigs (TCs)

^b ^number of sequences in TCs

^c ^number of unique sequences corresponding to wheat TFs in wDBTF

^d ^number of TFs with DNA-binding domain. For GRF, LUG, NZZ, Trihelix, ULT, Whirly, HRT-like and VOZ subfamilies no DNA-binding domain sequences are available in the Pfam database, so their attribution to a TF subfamily depended on their similarity to respective member sequences from rice database.

We chose to keep all sequences identified by their overall similarity with rice TF proteins in wDBTF and gave information on the positions of Pfam motifs on the table of subfamilies web page.

### Classification of wheat TF families

The wheat sequences were assigned to TF families by taking into account the best BLAST hit against rice TF proteins classified in the DRTF database http://drtf.cbi.pku.edu.cn/. In the wheat data set we found members corresponding to every rice TF family suggesting that TF families are conserved between rice and wheat. In wDBTF, wheat TF members were distributed across 40 families and 84 subfamilies. The NAC subfamily is the largest with at least 269 members. Among the other large TF subfamilies in wheat are the EREBP, AUX/IAA, bHLH, R2R3-MYB, bZIP, WRKY and C3H zinc finger families each with more than 150 members. At the other extreme, three subfamilies (LFY, SRS and LS) are each represented by a single gene. Wheat appears to have more AUX/IAA, CCATT-Dr1, HMG, MBF, S1Fa-like, Whirly and VOZ genes than would be predicted by comparing the number of genes proportionally to the number in rice, i.e. more than three times more. For each TF subfamily, a web page is available listing all accession numbers either for contigs or singletons.

### Wheat TF expression *in silico*

Almost 50% of sequences identified as wheat TFs are singletons and the majority (70%) of wheat TF contigs are made up of 2-5 ESTs. For all the families, the average number of ESTs per contig is 6.8. This suggests that wheat TFs, like other plant TFs, are not highly expressed.

By referring to the source of the material used to make each EST library, *in silico *expression data for each accession was compiled and is provided in the database.

We found 4,305 wDBTF wheat sequences are represented on the Affymetrix wheat genome array using Blast searches against the probe data sets. However, several sequences correspond to the same Affymetrix ID so only 2,036 are unique. This information was added to wDBTF as it could facilitate the study of families or organ-specific TF expression as the expression information relating to each probe can be analyzed across all publicly available Affymetrix results.

### Plant material for C2C2-Dof expression analysis

The wheat Dof TF sequences identified *in silico *were validated biologically by testing for their gene expression in seeds of *T. aestivum *line RE99006. Seeds were sown in 294-cm^3 ^pots filled with a peat moss mix and kept in a greenhouse for 2 weeks. After 8 weeks of vernalization, the plants were transplanted to soil beds in a greenhouse with daily maximum/minimum air temperatures averaging 21°C/15°C and day/night relative humidity averaging 50%/60%. At anthesis, spikes were tagged when the anthers of the central florets appeared and the temperature was monitored every 30 min by four HOBO^® ^H8 Indoor 8K Data Loggers (Onset Computer Corp., Bourne, MA). Two ears per plant were harvested every 50°C days from ovary stage until 600°C days after anthesis. All samples were taken at 11.00am to avoid possible diurnal effects on gene expression. Three independent biological replicates were used. The grains of central florets were collected, the embryo and the external pericarp were rapidly removed, and the endosperm was frozen in liquid nitrogen and stored at -80°C prior to RNA extraction.

### C2C2-Dof expression during seed development

Total RNA was extracted from ovaries and developing grains (without the embryo and external pericarp) as described in Khaled et al. [29]. Dof transcripts were amplified by PCR with an ABI Prism 7900HT sequence detection system (Applied Biosystems) using *Power *SYBR^® ^Green PCR Master Mix (Applied Biosystems) according to the manufacturer's instructions. Gene-specific primer pairs used for Dof amplification were designed with Oligo6^® ^and are listed in Additional file 2. Amplification of transcripts coding for actin, glyceraldehyde 3-phosphate dehydrogenase (GAPDH), elongation factor 1 alpha (eF1α), β-tubulin, and 18S was used for internal controls. The geometric mean was calculated and the normalized quantity of each gene transcript and standard deviation of the three replicates was then calculated [30]. All primer pairs gave an amplification efficiency of 100% ± 10% and were comparable. The specificity of amplification products was assessed by analyzing their melting curves and by gel electrophoresis (giving a single band). Amplification plots and predicted threshold cycle values were obtained from three independent biological replicates with the SDS software version 2.1 (Applied Biosystems).

## Utility

### wDBTF search

wDBTF is an integrated database which compiles all wheat sequences identified as TFs from publicly available expression data. Brought together in this form the data constitute a major resource for the study of gene expression in wheat and of plant TF families in general.

**Figure 3:**
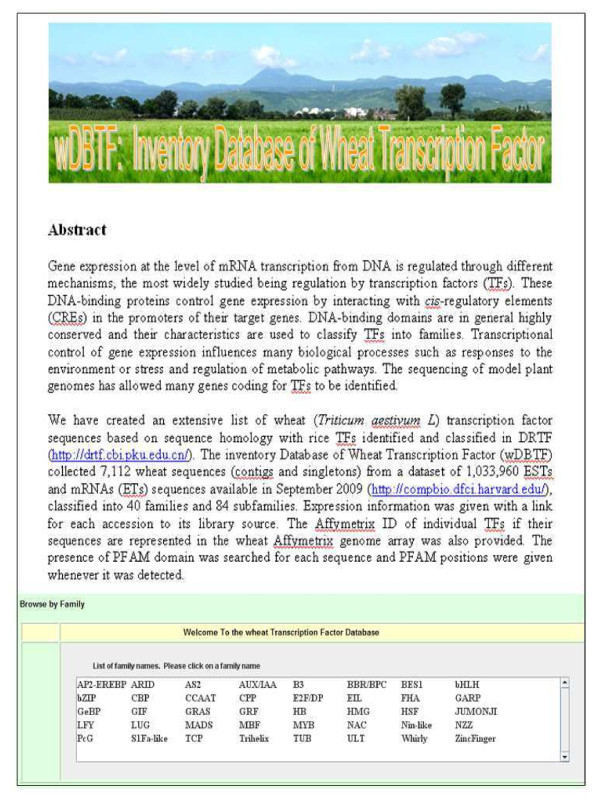
**Visualization of the wDBTF web interface main page**. The list of available families links to an individual page for each of the 84 analyzed TF subfamilies and then to the results page from which wheat sequences and additional information can be downloaded.

The wDBTF home page is the main web interface for all information contained in the database (Figure 3). The TF family names link to individual TF family or subfamily pages. Each TF family has its own dedicated page that contains 2 tables, either contigs or singletons, with all TF sequence accessions from the group.

The results page returns essential and comprehensive information, such as accession ID, best rice protein Blast hit, positions of Pfam domains and corresponding Affymetrix ID. *In silico *expression data is available for each accession referring to EST library sources. TF protein and DNA sequences and expression data can be downloaded from the wDBTF web site. Therefore, each family's results page integrates a large amount of data from multiple sources and is a good tool for research on the whole set of 7,112 TFs. Furthermore, classification associated with information on gene expression can be used to link candidate TFs to a given biological process.

### Analysis of C2C2-Dof family

**Figure 4:**
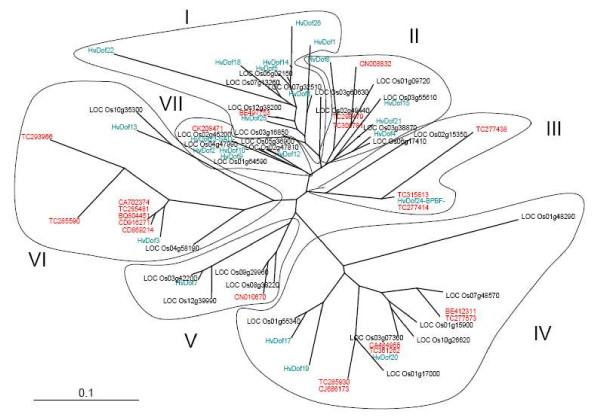
**Analysis of Dof family from wheat, barley and rice**. The accession numbers are indicated for *O. sativa *and *T. aestivum*. For *Hordeum vulgare *details were retrieved from Moreno-Risueno et al. [18]. The unrooted tree was created from an alignment of 50 amino acids of DOF domain sequences from three species using the neighbor-joining method. DOF from different species are shown in different colours (*T. aestivum *in red, *O. sativa *in black and *H. vulgare *in green). The seven groups are indicated and numbered. Scale bar = 0.1 substitution per site.

To illustrate our approach to *in silico *data analysis, we focused on the plant-specific Dof family. We identified 59 unique Dof sequences which are made of 186 expressed sequences in the above *T. aestivum *sequence output. In the wheat data set, 28 sequences cover Dof domains but only 22 cover it entirely. Dof domains of previously identified Dof genes from *O. sativa japonica *and *Hordeum vulgare*, described by Moreno-Risueno et al. [18], were downloaded from Genbank and were compared to the deduced amino acid sequences of the 22 wheat genes to establish relationships between the genes from different species. The conserved 50-amino acid Dof domains of the DOF proteins were aligned using CLUSTALW [31] and the resultant multiple alignment (Additional file 3) was represented as an unrooted neighbor-joining tree edited with the Treeview^© ^(v1.6.6) program [32] (Figure 4). Wheat Dof were present in the seven groups observed for the Dof family thus Dof members from the three cereals appeared in all the subfamilies independently of the species. Members were found for all major orthologous clusters (MCOG-A, -B, -C and -D) defined by Lijavetzky et al. [15]. Group I was shown to correspond to MCOG-B, group II to MCOG-A, groups III and VII to MCOG-C, and groups IV, V and VI to MCOG-D.

### Expression of *T. aestivum *Dof genes

We found 59 wheat Dof represented by 25 contigs and 34 singletons. Of the Dof contigs 60% are made up of 1-5 sequences, while only 5 have more than 10 ESTs indicative of high *in silico *expression.

**Figure 5:**
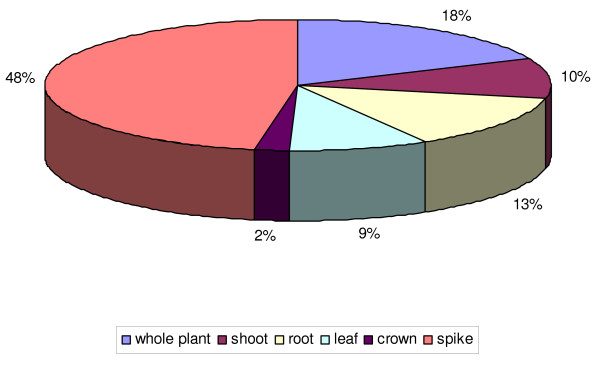
**Expression of Dof sequences in various organs of wheat**.

**Figure 6:**
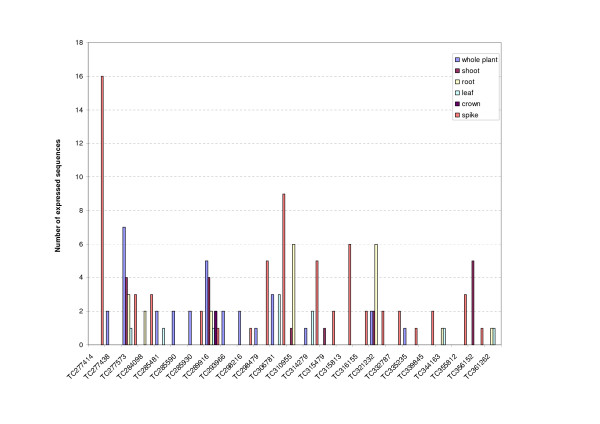
**Tissue specific *in silico *expression of wheat Dof as represented by EST contigs**.

**Figure 7:**
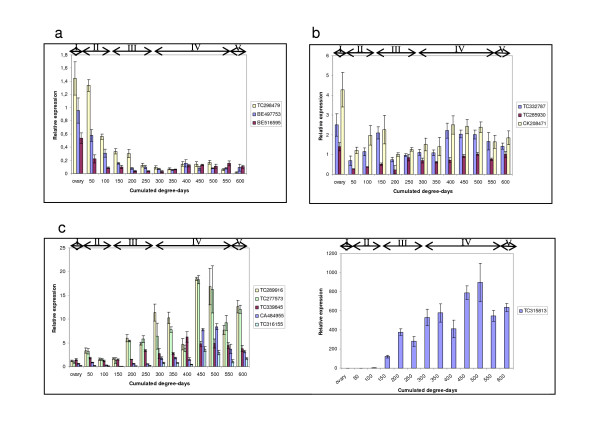
**Relative expression of twelve Dof genes in developing seeds of *T. aestivum *cv. RE99006**. Expression was monitored by qRT-PCR. Physiological stages of wheat seed formation: I, fertilization; II, cell expansion; III, cellularization and early grain filling; IV, completion of grain filling; V, desiccation. a) Expression pattern 1, b) expression pattern 2, c) expression pattern 3. TC315813 is considered separately because of its high expression level. Error bars represent the standard deviation of three replicates.

Of all the wheat Dof expressed sequences 48% come from spike libraries (Figure 5). *In silico *expression data show that 25 expressed Dof sequences from six contigs (Figure 6) and 19 singletons (Additional file 4) were detected only in spikes. Our primary focus is on identifying wheat Dof involved in endosperm development. cDNA made from RNA extracted from seed (300 degree-days after anthesis) of an INRA breeding line RE99006 was tested for the presence of 20 Dof transcripts using gene-specific PCR primers (Additional file 2); all these Dof transcripts were confirmed to be expressed in seeds (data not shown). To establish whether they are involved in wheat grain development, we tested the expression of 12 transcripts in endosperm at different seed developmental stages (Figure 7) by real time PCR. The same primers as above were used that gave efficient amplification and only one PCR-product in dissociation curves. Wheat Dof can be classified into 3 classes according to their expression profiles. Three Dof (TC298479, BE497753 and BE516595) are preferentially expressed during the first step of development when the grain is growing and their expression decreases down to a basal level in the grain-filling and drying-down stages. According to the second expression profile TC332787, TC285930 and CK208471 can be grouped together. These transcripts are detected through all developmental stages with three expression peaks: at the ovary stage, at early (150 degree-days) and mid grain-filling. Conversely to the first class, the third class groups Dof that are expressed at a basal level at the beginning of grain development increasing to a maximum midway through grain-filling when starch and storage proteins accumulate.

## Discussion

TFs are important for the regulation of eukaryotic gene expression. In constructing wDBTF the goal was to be comprehensive in collating both the wheat TF sequences and the information about each TF. Instead of relying entirely on computational prediction, we combined automated searches and manual curation to improve the quality of the database. In wDBTF, we define TFs as proteins that show sequence-specific DNA binding and are able to activate and/or repress transcription.

The large genome size of wheat (approximately 16,000 Mb) and its polyploid nature make the goal of sequencing the wheat genome a difficult one. ESTs are created by partially sequencing randomly isolated gene transcripts and they play an important role in gene discovery. The availability of more than 1 million *T. aestivum *ESTs derived from more than 250 libraries has proved an adequate resource for identifying wheat TFs with the added advantage that it can be used to identify tissue-specific expression patterns. In wDBTF, we considered 226 libraries that represent 98% of the publicly available wheat sequences of this kind.

The genomes of grasses are very different in terms of size, ploidy level and chromosome number. Despite these significant differences, the linear order of genes and protein coding sequences are very well conserved between genomes. Thus, the rice genome can serve as a good model for identifying genes in larger non sequenced genomes like the wheat genome. In addition, *O. sativa *TFs have been well defined [3,8] and information on these TFs has been organized into databases. Thus this species is ideally suited for comparative genomics like identifying wheat TFs. In the first step of our study, we used all TF protein sequences from rice DRTF as seed in Blast searches to better identify wheat TFs compared to using HMMER or core-consensus sequences. Significant similarity between known rice TF proteins and wheat accessions showed that 7,112 wheat accessions potentially belong to TF gene families. A minimum of 3,820 genes really corresponded to wheat TFs as they have a DNA-binding domain characteristic of a regulatory protein. Within the wheat data, we found members corresponding to every rice TF family suggesting that TF families are conserved between rice and wheat. Previous global approaches to studying plant TFs have already led to some TFs being identified from bread wheat ESTs [33]. PlantTFDB http://planttfdb.cbi.pku.edu.cn is a plant TF database for five model organisms, including Arabidopsis (DATF) and rice (DRTF), and 17 other plant species including wheat [33]. In October 2006 1,127 TFs were identified from wheat transcripts available in PlantGDB (v155a; http://www.plantgdb.org/) based on HMMER searches of the Pfam database (v20.0). These 1,127 TFs were classified into 57 families. For six families - GARP-ARR-B, HRT-like, LFY, NZZ, SRS, and ZF-HD - no TF was identified based on these criteria and no detail was given on whether allowance was made for the polyploidy of wheat. Two reasons could explain the differences between the results of this previous study and the work we report here. First, the results depend on the methods and cut-offs used, more or less stringent, to predict TFs. Depending on how the data is to be used, either high sensitivity or high specificity may be more desirable. Generally, stringent filtering is used to avoid false positives. To increase the chances of isolating TF genes from expressed sequences, especially for short or incomplete sequences and divergent members of gene families, we used less stringent criteria. Second, progress in generating DNA sequences in recent years has vastly increased the number of expressed sequences available in databanks to be searched. As the full genome sequence of wheat is not yet available, using all these sequences to update the catalogue of TF genes is useful.

To evaluate the accuracy and reliability of TFs identified in this way, we compared our results to other recent studies of wheat TF families. Using rice TF proteins from PlnTFDB v2.0 as seed in Tblastn searches, Shaw et al. [34] identified wheat Dof. Sequences containing the Dof domain were re-assembled with the criterion of 98% nucleotide identity thus defining 31 Dof genes. Similarly, we identified 28 Dof-domain genes using a different strategy based on another source of rice protein sequences. The differences observed may come from the way ESTs are assembled and from the discovery of a previously unpublished Dof member. Another TF family has been studied in detail by Stephenson et al. [12] who identified 37 CCAAT binding factors based in a Tblastn search of several EST sources (available in October 2006) with an e-value cutoff of 1e-08 using the core-conserved sequences of *O. sativa *and *A. thaliana*. With recent data and less stringent criteria we detected 81 CCAAT binding factors including those TFs previously detected. The results obtained with Dof and CCAAT TFs suggest that our approach performs well and with acceptable accuracy.

In plants, 5% to 7% of all the protein-encoding genes are for TFs [1,35]. Because many of the grass genomes are not yet completely sequenced or annotated, the total number of TFs expected is hard to predict. Based on the information available for four sequenced genomes, i.e. from Arabidopsis, rice, poplar (*Populus trichocarpa*), and Chlamydomonas (*Chlamydomonas reinhardtii*), linear regression was used to predict the number of TFs in non sequenced genomes from the total number of genes [36]. Studies based on the annotation of BAC sequences suggest that the hexaploid wheat genome could contain 108,000 genes [37] or between 164,000 and 334,000 protein-encoding genes including pseudogenes [38]. Thus, applying the linear regression to wheat would give 9,676 TFs. We have found a minimum of 3,820 sequences coding for TFs in data currently available, i.e. almost 40% of the expected number. However, 7,112 (about 73% of the expected number) wheat accessions have similarity with known rice TF proteins and could be potential TFs. Although further experimental evidence is needed to validate whether they really belong to TF families, we decided to keep all sequences in wDBTF so users can themselves judge the sequences with no DNA-binding domain.

The 3,820 wheat sequences identified as TFs represent 1.8% of all available unique wheat sequences. TF expression is mostly specific to a particular tissue or developmental stage and most TFs are also known to be expressed at a low level. We have shown that almost 50% of sequences identified as wheat TFs are singletons and 70% of contigs comprised just a few ESTs. This confirms that wheat TFs are expressed at a low level and suggests that perhaps not all TF sequences are represented in the EST libraries available. Therefore in our analysis of wheat expressed sequences, the number of TF genes could be underestimated. Until the whole genome sequence is available, it is unlikely that all the TFs can be identified based only on transcript sequences.

TFs have evolved by accumulating mutations, so the complexity in gene regulation patterns has increased. Much of the innovation in biological function has been attributed to events of gene duplication. Wheat appears to have more AUX/IAA, CCATT-Dr1, HMG, MBF, S1Fa-like, Whirly and VOZ compared with the predicted number, i.e. three times more than in rice. However, the largest families (EREBP, NAC, bHLH, R2R3-MYB, bZIP, WRKY and C3H zinc finger) do not exceed the proportional number predicted from rice. These preliminary results tend to confirm the coding theory that predicts that there are limits to the TF repertoire of cells. From each superfamily, the maximal number of TFs seems to correlate with the number of DNA bases effectively recognized by the binding mechanism of that superfamily. This limit of expansion is thought to minimize cross-binding errors between TFs [39].

Comprehensive Affymetrix GeneChip platforms have now been developed for wheat based on extensive EST collections http://www.plexdb.org/index.php. The Affymetrix wheat genome array can provide useful data on organ and developmental stage expression. We found 4,305 out of the 7,112 wDBTF wheat sequences represented on the Affymetrix wheat genome array. However, several sequences correspond to the same Affymetrix ID and only 2,036 are unique. Thus, this result is in agreement with those of Wan et al. [40] who found about 2,000 probe sets for potential TFs. Analysis of expression using this microarray platform can be difficult because of the often confounding presence of multiple splice forms, paralogs and orthologs, and, in the case of polyploids, homoeologs with near-identical sequences [41]. In general the wheat GeneChip is not able to distinguish contributions from individual homoeologs. As the probe sequences were specifically taken from conserved regions of consensus sequences, the expression profiles measured represent a sum of the individual profiles of the relevant homoeologs for ~90% of genes [42]. Furthermore, the expression measured could also be the sum of the expression of several splicing variants. TFs derived from the same precursor mRNA by alternative splicing may have distinct regulatory functions. In wheat, this kind of mRNA processing has been shown for some TF genes like the A and D copies of Viviparous 1 (TaVp1) [43] and for wDreb2 [44]. Parameters used in TIGR assembly often place homoeologous genes and alternative splicing variants in separate contigs and thus the difference observed between the number of sequences matching Affymetrix ID probes and the number of probes might be expected. On the other hand, singleton ESTs, relatively abundant in our study, tend to be shorter increasing the chances of confusion when trying to match them to a particular sequence present on GeneChip.

We used the Dof TF family to validate our strategy for identifying wheat TFs. Based on sequence identity with rice gene models, 59 potential Dof genes were identified. Multiple alignment of the entire Dof domain showed that wheat sequences were distributed in seven groups among all four major orthologous clusters suggesting this TF family is organized similarly in the species studied.

We used the database to select Dof involved in wheat grain development based on *in silico *expression criteria. First we verified these genes were indeed expressed in seed and then established the pattern of expression of 12 transcripts during seed development. For seven Dof (TC289916, TC277573, TC339845, CA484955, TC316155 and TC315813), expression reached a maximum in the middle of the grain-filling phase when starch and storage proteins accumulate. Accession TC315813 is wheat prolamin-box binding factor (WPBF), which was shown to be an activator of storage protein genes [24]. Its expression parallels the increase in abundance of transcripts encoding low molecular weight glutenin subunits and alpha gliadin genes consistent with a role in prolamin gene expression [40]. Thus, Dof TF expression profiles like that of *Wpbf *could be potential regulators of grain filling. In expressed sequences, there are few nucleotide variations between homoeologs. Thus the probability of designing primers specific to one or two copies is low. All the primers used amplified products from *T. urartu*, *Aegilops speltoides *and *A. taushii *which confirms that potentially all copies which are expressed in hexaploid wheat are amplified. Thus, the expression profiles measured for any particular gene may represent the expression of all its homoeologs. Plant responses to allopolyploidy include unequal expression of duplicated genes and gene silencing. Studies in wheat [45,46] have shown that novel patterns of gene expression occur in polyploids that are not observed in diploid progenitor species. Furthermore, *in silico *analysis of gene expression has shown that there may be preferential expression of homoeologous genes between genomes and a uniform level of expression of all three homoeologs is only observed for about 20% of the genes [45]. A recent study on the wheat TF *Spa *has demonstrated that the three copies were expressed during all stages of grain development in similar patterns. However, there was more *Spa *transcript from B genome than A and D transcripts [47]. To study patterns of gene expression in bread wheat, it will be essential to develop an assay that allows genome/homoeolog-specific gene expression to be quantified. Our preliminary results provide an overview of Dof expression and further research will confirm which homoeologous genes are implicated in the grain-filling mechanism.

## Conclusions

The genome sequence of wheat is not yet available so analyzing the systematically produced sequences of cDNA libraries is a powerful way to identify many transcripts that can then be studied in terms of their gene expression and functional genomics. In our study, wheat TF sequences were identified based on their similarity to those in the model plant rice. Organized in a dedicated database available on our web site, and classified in families are 7,112 potential unique wheat TFs, 3,820 of which have a DNA-binding domain. For each sequence, *in silico *expression data is given. The database will therefore be a major resource for the plant research community for future studies of wheat TFs.

## Availability and requirements

The wheat transcription factor inventory database wDBTF is now available at http://wwwappli.nantes.inra.fr:8180/wDBFT/

License: none.

Any restrictions to use by non-academics: none.

## Authors' contributions

IR analyzed data, designed and assessed specific primers for Dof transcript amplification and wrote the manuscript. DT has developed bioinformatics tools. MD carried out the RNA extraction and PCR amplification. CR performed the Blast searches against Affymetrix probes, participated in coordination and in the design of the study and helped to draft the manuscript. GB and GC participated in coordination and helped to draft the manuscript. All authors read and approved the final manuscript.

## Supplementary Material

Additional file 1 **The 226 wheat ESTs libraries used for TF analysis**. A table showing banks used for this database construction and corresponding tissue and EST number. Click here for file

Additional file 2 **Sequences of primers used for detection of Dof transcripts**. A table of sequence accession names and DNA probe sequences of forward and reverse primers used for Dof transcripts PCR amplification. Click here for file

Additional file 3 **Multiple sequence alignment of the Dof DNA-binding domain from wheat, rice and barley**. A protein sequence alignment of the Dof domain resulting from CLUSTALW. The levels of amino acid conservation at each position among the Dof members are indicated in the first histogram with the highest bars representing 100% amino acid identity. The black histogram represents the consensus amino acid sequence. Click here for file

Additional file 4 **Tissue-specific *in silico *expression of wheat Dof singletons**. A table showing EST accession names of wheat Dof singletons and their corresponding tissue of origin. Click here for file
