# Supplementary material for: wDBTF: an integrated database resource for studying wheat transcription factor families
Source: BMC Genomics. 2010 Mar 18;11:185. doi: 10.1186/1471-2164-11-185 (PMC2858749; doi:10.1186/1471-2164-11-185)
Supplement: Additional file 1 — The 226 wheat ESTs libraries used for TF analysis. A table showing banks used for this database construction and corresponding tissue and EST number. [file 1471-2164-11-185-S1.PDF]

Bank	Tissue	EST number
wws	scutellum	1076
psrp	pericarp	571
e411	maternal tissue	568
e512	maternal tissue	618
o232	aleurone	747
d15	embryo	932
d26	embryo	440
d37	embryo	480
wem1c	embryo	249
j122	embryo	927
j223	embryo	958
j324	embryo	514
l125	embryo	579
l226	embryo	748
n129	embryo	948
aba	embryo	2173
wedorem	embryo	2954
n130	embryo	1013
wde1f	endosperm	738
e29	endosperm	749
we610	endosperm	2564
e310	endosperm	753
we612	endosperm	4433
h116	endosperm	849
wde2f	endosperm	664
whe	endosperm	2139
we530	endosperm	2824
psre	endosperm	230
scu	endosperm	1152
wemat	endosperm	1216
gr45	seed	7956
wdk1c	seed	4860
tae05	seed	5881
whms	seed	10729
whokcs	seed	10461
g118	seed	9830
wdk2c	seed	5017
wheYO	seed	11282
g174	seed	5165
wdk3c	seed	5054
tae15	seed	5496
g356	seed	4249
wdk9n	seed	331
whdpa20	seed	11308
wdk4c	seed	971
g468	seed	5802
g550	seed	5951
whsYO	seed	12572
wdk5c	seed	854
g608	seed	5261

g750	seed	4864
wg344	seed	3649
whemi	seed	10217
whemc	seed	10210
whem	seed	10155
whoh	pistil	10349
whgcpcdam	anther	9424
whpcdam	anther	9205
whmeiotic	anther	9139
whhgcpcdam	anther	9008
wov	ovary	5311
wpa1c	anther	4858
wlp1c	lemma	2306
wip1c	pistil	1955
wfloret	florets	1501
waw1c	anther	1475
awb	florets	988
a11	carpel	786
wia1c	anther	775
q343	ovary	767
q141	ovary	701
q242	egg cell	654
flA22	carpel	476
fla22b	carpel	365
whfl	spikelet	24568
whspi	spikelet	14770
wpreanth	spike	13397
whf_spi	spike	12300
whyd	spikelet	12030
whh	spike	11285
whyf	spikelet	11072
wfusspi	spike	11045
wspike5	spike	3454
wmug	spikelet	2242
wdi1c	inflorescence	2201
ta04	heads	1724
ta03	heads	1697
ta01	heads	1586
ta08	spikelet	1532
wheatspi	spike	1275
ta02	heads	1230
ta09	spikelet	1024
wet2s	inflorescence	1021
wet1s	inflorescence	942
ta10c	spikelet	895
spikecs	spike	871
spikeksu	spike	727
sumai3	spike	418
ta10b	spikelet	358
ta07b	spikelet	281
aafc	spike	242

wet1c	inflorescence	227
spikecs515	spike	115
wl1n	leaf	11100
wlm96	leaf	10915
wle1n	leaf	8888
TaLr1	leaf	7460
wlsu2	leaf	5253
F1	leaf	4923
wlm0	leaf	4551
wlsu1	leaf	4392
AZO3	leaf	3757
AZO1	leaf	3105
wlmk1	leaf	2974
wri1s	leaf	2929
wpi2s	leaf	2761
wlm24	leaf	2428
wlk1	leaf	2243
wlm1	leaf	2207
wri2s	leaf	2041
wlm12	leaf	1934
wlmk4	leaf	1822
wlk8	leaf	1741
wlk4	leaf	1591
wlm4	leaf	1496
wlmk8	leaf	1482
ITEC_SCL	leaf	1002
wl1	leaf	688
wpi1s	leaf	2675
csdroughts	leaf	790
cnw	leaf	766
zap	leaf	532
jjl	leaf	449
wle1n	leaf	388
fidel	leaf	335
wfl1c	leaf	217
whkmp	shoot	11354
whkmv	shoot	10742
whshc	shoot	10658
whvwd	shoot	10395
whshdr	shoot	9887
v4816	shoot	9768
v483	shoot	9183
whvhs	shoot	9102
whvaba	shoot	9042
w4etiol	shoot	4128
wstemunst	stem	2457
wstemunstN	stem	3490
wstemhessian	stem	919
wstemetiol	stem	332
wre1n	root	16792
wr1	root	14967

FGAS	root	13098
whr	root	10502
AZO2	root	8949
wheat_etiol	root	4596
wheat_etiol_norm	root	4596
AZO4	root	2876
whrdr	root	10635
wrootsalt	root	2055
wrootdrought	root	1310
wswr	root	1274
mwl	root	1038
walu	root	1032
whatlal	root	25819
whsct	root	25616
whsctal	root	24171
whatlal	root	23879
wrootunst	root	1025
walust	root	991
wrootunst2	root	959
wdr1f	root	933
p436	root	715
csrootetiol	root	697
wrsu1	root	509
wre1n	root	455
p537	root	448
wwr	root	434
p840	root	420
gbx	root	322
azo5	root	194
csrootetiol2	root	169
whdl	crown	10069
fgas7	crown	4532
fgas6	crown	4093
fgas5	crown	3639
fgas4	crown	3184
wvern	crown	2785
fgas2	crown	2492
fgas3	crown	1739
wsaltcrown	crown	1183
crownssv	crown	204
crownssh	crown	171
whcsec	callus	10216
coldTreated	whole	1216
Cswhole	whole	1404
cultcell	culture cell	10805
fgasl2g3	mixted	28152
fgasl4g8	mixted	3086
fgasl5g7	mixted	7131
fgasl6g1	mixted	9675
p234	whole	1265
T06	whole	1022

wcoldStressed	whole	1188
wds1c	whole	1051
wds3f	whole	1029
wh_sh	whole	6278
whchan	whole	24162
whchul	whole	24020
whthkles	whole	23503
whthls	whole	23683
wyr1c	whole	1550
ta07	whole	1002
p638	whole	945
p335	whole	925
p133	whole	907
ta05	whole	902
wholedrought	whole	879
wds1f	whole	826
wholecs	whole	687
p739	whole	662
unspec	whole	1030
unspec2	whole	762
whole2045	whole	1208
wholesub	whole	1000
wdr1	whole	400
	**Total**	1036933
