# Supplementary material for: wDBTF: an integrated database resource for studying wheat transcription factor families
Source: BMC Genomics. 2010 Mar 18;11:185. doi: 10.1186/1471-2164-11-185 (PMC2858749; doi:10.1186/1471-2164-11-185)
Supplement: Additional file 2 — Sequences of primers used for detection of Dof transcripts. A table of sequence accession names and DNA probe sequences of forward and reverse primers used for Dof transcripts PCR amplification. [file 1471-2164-11-185-S2.PDF]

TIGR accession	forward primer	reverse primer
BE403347	CGACTTTGATGCCATGAG	GGGAAGCCTTGCATCT
BE497753	CCGCGGCCGTTAGTG	TGGCGCGACGACTTCT
CN008832	CTGCGAGTCCACCGA	TGCGAGAGGTTGTAGTTG
CN010670	GGGACACCAAGTTCTGCT	GGCTGGAGGACGAGGA
TC332787	CGCGTTCGAGGACTTG	GCCGATCATGCCGTT
TC321232	CCACCGCCTACGAGAT	CGTAGAAGCCGTCAAAG
TC356152	TGAGTGTTGCCGACCA	GATCCGCAGAGACGAAG
CK208471	CGGCTCACGGAAGAAC	CGGACGGGTGCAAGA
BE516595	CTGTTGCCATCCTACCA	GCTGTTCCCGTTCGAG
AL814118	TGCCGATCATGACTGG	ACTTCATTACCGCCTCA
TC298479	TCCTCCCGCTCCAACA	CCTGGTCGGCGAATATC
TC285930	CCTGATATAATCGTGCCAT	TCCAGGAGCAGCTATGTTA
TC316155	GATGGCAAAGGGTGTAAG	AGGCAGCATGAAGAATTT
CA484955	GAAACAGAGCGGCTTTG	GATGGTCTTCCCGAACA
TC289916	ACCTCCCACAAGCAGTAGT	ACTCTTTGCAGCTTCGTC
TC277573	TTCGAGTGCGTGTGAGA	TGCCGTCCAATACCTTT
TC339845	CGCAGTTCGACCTCG	ACGCCGATCATCAGTT
CA628527	GCCAGTAGCAACATCAAC	TGCGAACGTCGAAATGT
CA699681	TGAACTCTCCCATGATGC	GACCCAGACGGAGATATG
TC315813	AGAAGAAGCCTCGGCCAAAG	CAGAACTTGGTGTTGCCAGACTT

Gene	forward primer	reverse primer
GAPDH	TTCAACATCATTCCAAGCAGC	CGTAACCCAAAATGCCCTTG
eF1α	CAGATTGGCAACGGCTACG	CGGACAGCAAAACGACCAAG
β-tubulin	CCATCAGTTGGTTGAGAATGC	CAAAGCTGGGAGTGGTCA
18S	CCATCCCTCCTCCGTAGTTAGCTTCT	CCTGTCGGCCAAGGCTATATAC
