# Supplementary material for: wDBTF: an integrated database resource for studying wheat transcription factor families
Source: BMC Genomics. 2010 Mar 18;11:185. doi: 10.1186/1471-2164-11-185 (PMC2858749; doi:10.1186/1471-2164-11-185)
Supplement: Additional file 3 — Multiple sequence alignment of the Dof DNA-binding domain from wheat, rice and barley. A protein sequence alignment of the Dof domain resulting from CLUSTALW. The levels of amino acid conservation at each position among the Dof members are indicated in the first histogram with the highest bars representing 100% amino acid identity. The black histogram represents the consensus amino acid sequence. [file 1471-2164-11-185-S3.PDF]

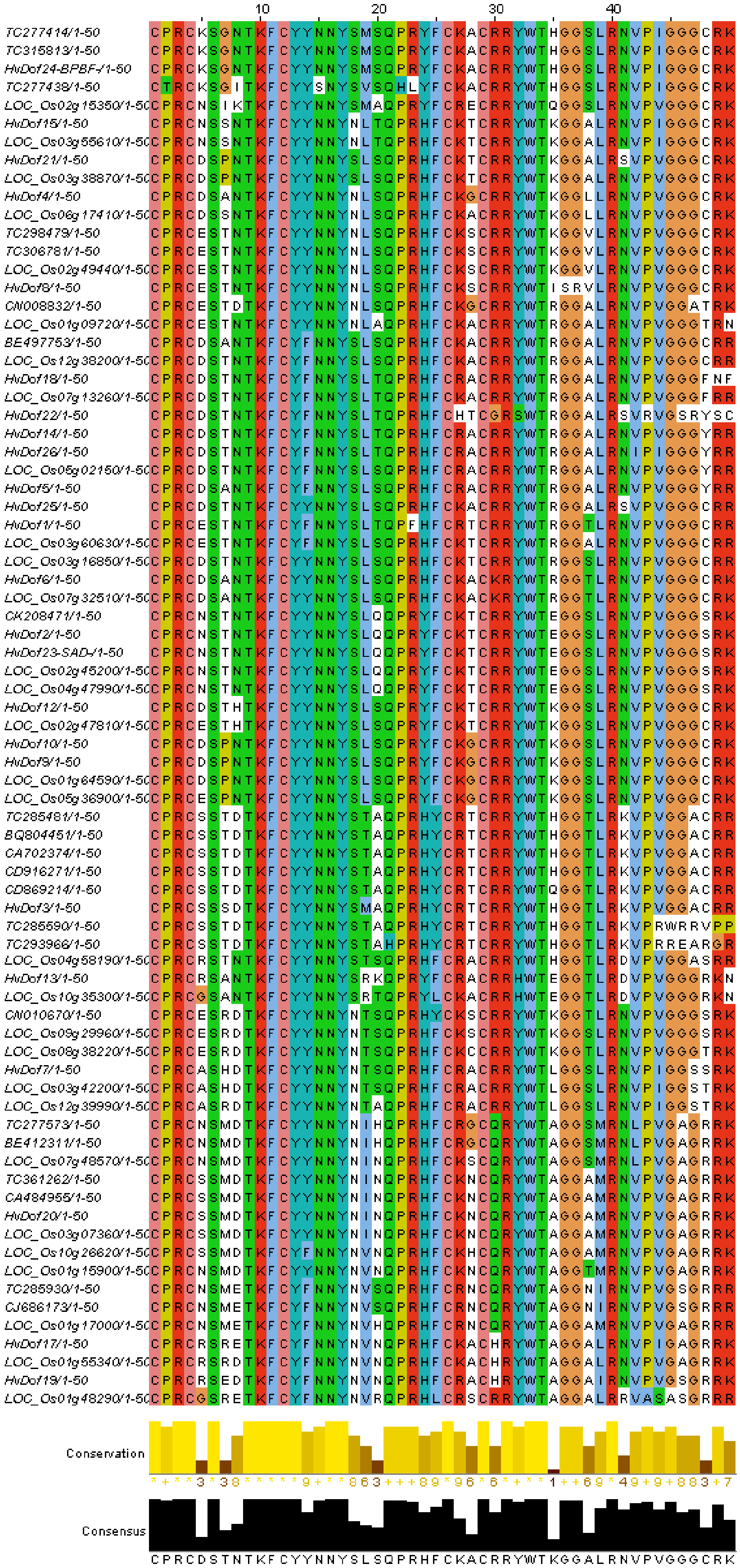

10
20
30
40
Conservation
Consensus
CPRCDSTNTKFCYYNNYSLSQPRHFCKACRRYWTKGGALRNVPVGGGCRK
