# Supplementary material for: wDBTF: an integrated database resource for studying wheat transcription factor families
Source: BMC Genomics. 2010 Mar 18;11:185. doi: 10.1186/1471-2164-11-185 (PMC2858749; doi:10.1186/1471-2164-11-185)
Supplement: Additional file 4 — Tissue-specific in silico expression of wheat Dof singletons. A table showing EST accession names of wheat Dof singletons and their corresponding tissue of origin. [file 1471-2164-11-185-S4.PDF]

Origin of EST	Sequences name
whole plant	CK208471;CK212053;CV770319
shoot	CJ616808
root	BE403347;CD869214;DR734966
leaf	BE412311;CA619223;CA622030;CA628527;CA660010;CA699681
crown	CK154911;CK155202
Spike	AL814118;BE497753;BE516595;BQ608548;BQ804451;CA484955; CA485059;CA485447;CA593327;CA594242;CA702359;CA702374; CA705158;CA733531;CD916271;CD931123;CJ686173;CN008832; CN010670
